# Genomic Selection and Association Mapping in Rice (*Oryza sativa*): Effect of Trait Genetic Architecture, Training Population Composition, Marker Number and Statistical Model on Accuracy of Rice Genomic Selection in Elite, Tropical Rice Breeding Lines

**DOI:** 10.1371/journal.pgen.1004982

**Published:** 2015-02-17

**Authors:** Jennifer Spindel, Hasina Begum, Deniz Akdemir, Parminder Virk, Bertrand Collard, Edilberto Redoña, Gary Atlin, Jean-Luc Jannink, Susan R. McCouch

**Affiliations:** 1 Department of Plant Breeding and Genetics, Cornell University, Ithaca, New York, United States of America; 2 International Rice Research Institute, Los Baños, Philippines; 3 International Center for Tropical Agriculture, Cali, Colombia; 4 Bill & Melinda Gates Foundation, Seattle, Washington, United States of America; 5 US Department of Agriculture—Agricultural Research Service (USDA-ARS), Ithaca, New York, United States of America; University of Georgia, UNITED STATES

## Abstract

Genomic Selection (GS) is a new breeding method in which genome-wide markers are used to predict the breeding value of individuals in a breeding population. GS has been shown to improve breeding efficiency in dairy cattle and several crop plant species, and here we evaluate for the first time its efficacy for breeding inbred lines of rice. We performed a genome-wide association study (GWAS) in conjunction with five-fold GS cross-validation on a population of 363 elite breeding lines from the International Rice Research Institute's (IRRI) irrigated rice breeding program and herein report the GS results. The population was genotyped with 73,147 markers using genotyping-by-sequencing. The training population, statistical method used to build the GS model, number of markers, and trait were varied to determine their effect on prediction accuracy. For all three traits, genomic prediction models outperformed prediction based on pedigree records alone. Prediction accuracies ranged from 0.31 and 0.34 for grain yield and plant height to 0.63 for flowering time. Analyses using subsets of the full marker set suggest that using one marker every 0.2 cM is sufficient for genomic selection in this collection of rice breeding materials. RR-BLUP was the best performing statistical method for grain yield where no large effect QTL were detected by GWAS, while for flowering time, where a single very large effect QTL was detected, the non-GS multiple linear regression method outperformed GS models. For plant height, in which four mid-sized QTL were identified by GWAS, random forest produced the most consistently accurate GS models. Our results suggest that GS, informed by GWAS interpretations of genetic architecture and population structure, could become an effective tool for increasing the efficiency of rice breeding as the costs of genotyping continue to decline.

## Author Summary

Genomic selection is a promising breeding technique that aims to improve the efficiency and speed of the breeding process. While it has been shown to be effective in crops such as wheat and corn, it has not yet been applied to rice breeding. Genome-wide association studies (GWAS), by contrast, are used to identify genes or QTLs that underlie traits of importance to breeding such as yield, flowering time, or plant height, and has been performed successfully in rice. Here, we experiment with applying genomic selection in conjunction with GWAS to a rice breeding program at the International Rice Research Institute in the Philippines and show that genomic selection can result in more accurate predictions of breeding line performance than pedigree data alone and that GWAS results can inform the results of GS. Our results suggest that GS could be an effective tool for increasing the efficiency of rice breeding.

## Introduction

Over the next 30 years, the production of staple cereal grains including wheat, maize, and rice must to be doubled to keep pace with global population and income growth. At the same time, agriculture, in general, is imperiled by human-induced climate change, and plant breeders and farmers together must contend with increased biotic and abiotic stresses that are the direct result of climate unpredictability. Breeding rice varieties adapted to the Asian tropics is already a challenging and resource-intesive endeavor. The number of bacterial, fungal, viral and insect pests for tropical irrigated rice outnumber those for other major cereals. For non-irrigated rice, abiotic stresses such as flooding and drought also negatively affect production [1,2,3]. Rice breeders must therefore consider a large number of simple and quantitative traits in combination when developing new lines while, at the same time, maintaining and improving quality and ensuring yield improvements over existing varieties. Using coventional breeding methods, this process is extremely time consuming—on average, it takes up to ten years for elite varieties to be developed and identified [4].

The majority of public sector rice breeding programs in Asia still use conventional breeding schemes. By far, the most common way of breeding is the pedigree method, which involves visual selection and trait screening over several successive generations [2]. With advances in rice molecular genetics and genomics, however, other potentially faster breeding methods are being developed. Marker assisted selection (MAS), in which a small number of molelcular markers are used to tag genes-of-interest, has been implemented for rice improvement, but its overall impact on enhancing the efficiency of breeding has been limited [5]. MAS has been successfully used in rice to incorporate major genes and/or large-effect quantitative trait loci (QTLs) controlling abiotic stresses such as submergence, salinity and drought into new varieties [6]. However, most traits of interest to rice breeders are not controlled by just a few large-effect genes, but by many genes of small effect and/or by a combination of major and minor genes. MAS is far less suitable for these types of trait genetic architectures, so its utility to rice breeders is limited. Epistatic interactions and the effects of genetic background in rice furthermore make molecular breeding even more complicated.

Genomic selection (GS), introduced in 2001 by Meuwissen and colleagues, presents a new alternative to traditional MAS that has enormous potential to actually improve gain per selection in a breeding program per unit time, and thus breeding efficiency. In a GS breeding schema, genome-wide DNA markers are used to predict which individuals in a breeding population are most valuable as parents of the next generation of offspring [7]. These estimated values, termed the genome estimated breeding values (GEBVs), are output from a model of the relationship between the genome-wide markers and phenotypes of the individuals undergoing selection. The GEBVs are then used to select the best parents for making new crosses. The GS model itself is developed from a training population that resembles the population under selection (also referred to as the testing population); it is both genotyped and phenotyped, while the testing population is genotyped only. The testing population genotypes are then entered into the model to calculate the GEBVs of all the individuals in the population, even those that have not been phenotyped. Thus, the key difference between GS and traditional MAS is that genotyping is not limited to a selected set of markers that tag putative genes, but rather breeding value is predicted based on all available marker data to avoid ascertainment bias and information loss. Including all markers in the model regardless of effect size also makes it possible for the first time to track and select for small effect genes/QTL in addition to large effect genes/QTL. Statistical shrinkage, Bayesian, and/or machine learning methods are used to fit the many thousands of effects [7,8].

The advantage of GS over the widely-used traditional pedigree breeding method is thus one of breeding efficiency. Gain from selection during GS is proportional to GEBV accuracy. As a result, when GEBV accuracy is high enough, GS can reduce breeding time by increasing the proportion of high-performing offspring in a breeding population, thus accelerating gain from selection [9,10]. GS has been most successfully implemented in dairy cattle breeding, where its efficiency is proven: the replacement of progeny testing with the genotyping of young bulls has cut generation interval time in half [11]. Genetic heterogeneity is also low for Holstein-Friesian cattle, and GxG and GxE effects are limited, which makes prediction of breeding value simpler [5]. In plant breeding, these interactions present a challenge, as does the presence of structure within and between breeding populations, but GS still holds the potential to improve breeding efficiency. In temperate crops GS can accelerate gain from selection per unit time beyond that gained by the overall population improvement described above through the use of off-season nurseries [12,13], while in tropical crops like rice, GS can be used with one or more cycles of rapid generation advance [14] for a similar gain.

In plants most applied GS experiments to date have been in maize and small grains, and it is quickly generating interest as a breeding tool for those crops. High GEBV accuracies for grain yield and a variety of other quantitative traits have been obtained for both maize and wheat bi-parental and double haploid populations using experimental cross-validation [15,16], and GS has been demonstrated to outperform marker assisted recurrent selection (MARS) in at least one maize breeding program [17]. Moderate cross-validation prediction accuracies have also been obtained for yield and a variety of other traits in diverse germplasm collections and breeding populations of maize, wheat, oat, and barley [18,19,20,21,22,23]. Preliminary genomic selection research has also been published on several other crop plants including cassava, sugarcane, and sugar beet [24,25,26,27].

Several recent studies in maize, however, advise caution regarding the presence of hidden or known structure or family relatedness within a breeding population or germplasm collection when estimating GS accuracy. Windhausen *et al* (2012) found that within a diversity panel of 255 maize lines from eight distinct breeding populations any predictive ability in the dataset was a byproduct of the population structure, while Riedelsheimer *et al* (2013) found mean accuracies of 0% when trying to predict individuals in biparental families using data trained on the progeny of an unrelated cross [28,29]. To accurately predict the phenotypes of individuals in their biparental crosses, Riedelsheimer *et al* found it necessary to train the model using full sibs of the validation individuals, or half sibs representing both parents of the cross [28].

These limitations will likely also apply to rice, which is subject to deep population structure and is often bred in large, inter-related pedigree schemas. Rice is also frequently admixed, and many varieties contain introgressions from different subpopulations [30,31,32]. The work of Guo *et al*., (2014) evidences this need to control for subpopulation structure when performing GS in rice. In a rice diversity panel, Guo *et al*. (2014) found that ∼33% of the genomic heritability was explained by subpopulation structure, and that controlling for subpopulation structure when performing cross-validation significantly decreased prediction accuracy. When prediction was performed within a specific subpopulation, however, structure was found to have little effect on prediction accuracy [33]. This is fortunate for breeding programs, which generally work within a particular subpopulation, although introgressions are frequent.

Genetic architecture must also be taken into account when considering the implementation of genomic selection. GWAS results in maize have consistently found most agronomic traits to be controlled by many genes of small effect [34,35,36,37]. In rice, by contrast, many GWAS and QTL mapping studies have found large effect QTLs for agronomic traits, including grain yield, flowering time, plant height, aluminum tolerance, grain yield under drought stress, and submergence tolerance [38,39,40,41,42,43,44]. The difference in the genetic architecture between maize and rice, as well as the difference in the genetic architecture of different rice traits, could be expected to affect the relative efficacy of different genomic selection statistical methods.

To the best of our knowledge, no research on performing GS in a rice breeding population has yet been published. Here we report the results of performing GWAS and GS cross-validation using data on a collection of 363 elite breeding lines from the International Rice Research Institute's (IRRI's) irrigated rice breeding program. To assess GS accuracy, we performed five-fold cross validation to predict grain yield, flowering time, and plant height in the 2012 wet and dry season in Los Baños, Philippines and compared our prediction results using GS to those using only pedigree information as well as a traditional MAS model. For the GS models, the training population composition, marker number, and the statistical method for the calculation of GEBVs were varied to determine their effect on rice GS accuracy. Finally the GWAS results published in a companion paper allowed us to analyze the effect of genetic architecture on GS prediction accuracy [45].

## Results and Discussion

### Genotyping and fold design

384-plex Genotyping-by-sequencing (GBS) was used to discover and call SNPs on 369 advanced inbred breeding lines from IRRI's irrigated rice breeding program (methods). SNP calling was performed using the TASSEL3.0 GBS pipeline with physical alignment to the MSU v6.0 Nipponbare rice reference genome using Bowtie2 [46,47]. The resulting SNP data were imputed using the TASSEL 3.0 FastImputationBitFixedWindow plugin [48]. After imputation, SNPs with call rates < 90% were removed along with monomorphic markers to obtain a filtered SNP dataset containing 73,147 SNPs. Individuals with missing data > = 60%, a total of six individuals, were dropped for a total of 363 genotyped lines (materials and methods).

The majority of the 363 lines were known *a priori* from breeding records to belong to the *indica* or *indica-admixed* subpopulation groups. In order to identify outlier individuals belonging to the *japonica* or *japonica-admixed* groups, principle components analysis (PCA) was performed using the 73,147 SNPs. Thirty one outliers were identified and excluded based on this analysis (S1 Fig.). After removing these 31 outliers, the resulting PCA suggested no remaining subpopulation stratification within the dataset. Family structure, however, was presumed to still exist. As the presence of close relatives (e.g. full sibs) across training and testing folds in a cross-validation experiment can artificially inflate prediction accuracies, it was necessary to also control for this family structure. To do so, the remaining 332 lines were clustered using a partitioning around k-medoids algorithm (PAMK) based on the genotype matrix. k = 87 was found to be the most statistically favorable number of clusters in the dataset based on average silhouette width (S2 Fig.). Individuals in the same cluster (of 87) were then assigned to the same fold of 5 to form the five folds used for cross-validation. The most closely related individuals were thus placed within the same fold, making it impossible for them to be spread across training and testing groups [26] (materials and methods).

### Phenotypes

Four years of grain yield (kg/ha), flowering time (days to 50% flowering), and plant height (cm) data and related phenotypic covariates were curated from historical breeding trial records taken at a single location in Los Baños, Philippines for years 2009–2012, two seasons per year, dry and wet, with the exception of plant height in the 2009 wet season, which was not available (materials and methods). As the genotyped lines represent a subset of a working breeding program, substantial missing data are present in years 2009–2010 for all traits (S1 Table). Such an unbalanced design is typical of breeding trial data and to be expected in the practical implementation of GS. Correlations among years/seasons were calculated for all three traits using the trait least squares means. For grain yield, the 2011 and 2012 data were more tightly correlated than the earlier year data. Flowering time and plant height data was well correlated for all four years and seasons (S3 Fig.) (see methods).

Narrow-sense heritabilities were calculated on a per line basis for each trait for both validation seasons—the 2012 dry season (2012 DS) and the 2012 wet season (2012 WS) and ranged from 0.31–0.32 for grain yield, 0.30–0.35 for plant height, and 0.32–0.44 for flowering time (Table 1) (materials and methods). Heritabilities for all three traits were slightly higher in the dry season than the wet season.

**Table 1:** Trait heritabilities.

Trait	Season	h^2^
YLD	DS 2012	0.3213
PH	DS 2012	0.3546
FL	DS 2012	0.4378
YLD	WS 2012	0.3059
PH	WS 2012	0.3036
FL	WS 2012	0.3254

Narrow-sense heritabilities (h^2^) for the two validation season, 2012 dry season (DS 2012) and the 2012 wet season (WS 2012). YLD = grain yield, FL = days to 50% flowering, PH = plant height.

### Cross validation using 73,147 markers

Five-fold cross validation was performed using the full set of 73,147 markers to predict grain yield, flowering time, and plant height in the 2012 dry and wet seasons. The year and season data included in the training population were varied to determine which combinations of years/seasons were the most predictive of the 2012 dry and wet season (total of twelve different combinations). The GEBV accuracies were calculated as the correlation of predicted GEBV and observed phenotypes in the validation population.

Six statistical methods widely demonstrated to produce accurate genomics-assisted breeding models in a variety of crops were selected from the literature to test using our rice data. The selected methodologies were chosen to represent the variety of available approaches, and included one linear, parametric, and frequentist method: rrBLUP, one linear, parametric, and Bayesian method: Bayesian LASSO (BL), one non-linear semi-parametric method: Reproducing Kernel Hilbert Spaces (RKHS), and one non-linear machine learning method: Random Forest (RF) [19,23,49,50]. Multiple Linear Regression (MLR), in which a subset of significant markers are chosen to fit a linear model, has been shown to be effective for traits with a very simple genetic architecture, and served as our non-GS method control [51]. Finally, kinship BLUP was used to predict GEBV based on the pedigree A-matrix alone (ped) (methods) [52].

We estimated accuracies using three experiment types (CV1, CV2, and CV3). CV1 accuracies were calculated using training populations that included data from the validation year/season, i.e, if the validation population consisted of the 2012 dry season, then data on individuals from the 2012 dry season were included in the training population, excluding data on any individuals in the validation fold. However, this is likely to upwardly bias accuracy estimates by confounding GxE and line effects [21], so we worked to obtain an estimate of this bias by performing two other types of experiments. For CV2 accuracies we excluded the validation year/season from the training population. By removing these data from the training population, however, we introduce a different confounding factor to our accuracy estimate—a smaller training population size. We therefore performed cross validation experiment 3 (CV3) in which the data from the validation year/season were retained in the training population, but the equivalent data from the respective 2011 season were *not* included in the training population. The overall estimate of bias for a given permutation was subsequently estimated as accuracy of CV3—accuracy of CV2 [26] (materials and methods). The bias estimates were found to be very small and consistent for all tested traits and permutations (Table 2, S2–S4 Tables). It can thus be concluded that for the population and statistical methods tested here bias as a result of including data from the validation year/season in the training population is not a significant concern.

**Table 2:** Summary of best performing GS experiments for predicting grain yield (YLD), flowering time (FL), and plant height (PH) in the 2012 dry season (2012 DS) and the 2012 WS (2012 WS)

Trait	TP	VP	stat method	accuracy
YLD	2009–2011 all	2012 DS	^A^ RR-BLUP	0.3044
YLD	2009–2011 all	2012 DS	^A^ RKHS	0.2596
YLD	2009–2011 all	2012 DS	^A^ RF	0.2458
YLD	2009–2011 all	2012 DS	^B^ ped	0.2146
YLD	2009–2011 all	2012 DS	^C^ BL	0.1358
YLD	2009–2011 all	2012 DS	^D^ MLR	-0.0599
YLD	2009–2011 all	2012 WS	^A^ RF	0.3136
YLD	2009–2011 all	2012 WS	^A^ RR-BLUP	0.2852
YLD	2009–2011 all	2012 WS	^A^ RKHS	0.2399
YLD	2009–2011 all	2012 WS	^B^ ped	0.1904
YLD	2009–2011 all	2012 WS	^C^ BL	0.0876
YLD	2009–2011 all	2012 WS	^D^ MLR	0.0095
FL	2009–2011 DS only	2012 DS	^D^ MLR	0.6270
FL	2009–2011 DS only	2012 DS	^A^ RF	0.6093
FL	2009–2011 DS only	2012 DS	^A^ RR-BLUP	0.4919
FL	2009–2011 DS only	2012 DS	^A^ RKHS	0.4865
FL	2009–2011 DS only	2012 DS	^C^ BL	0.4536
FL	2009–2011 DS only	2012 DS	^B^ ped	0.3997
FL	2010–2011 all	2012 WS	^D^ MLR	0.5400
FL	2010–2011 all	2012 WS	^A^ RF	0.4187
FL	2010–2011 all	2012 WS	^A^ RKHS	0.3872
FL	2010–2011 all	2012 WS	^A^ RR-BLUP	0.3808
FL	2010–2011 all	2012 WS	^C^ BL	0.3237
FL	2010–2011 all	2012 WS	^B^ ped	0.2071
PH	2009–2011 DS only	2012 DS	^A^ RF	0.3411
PH	2009–2011 DS only	2012 DS	^A^ RR-BLUP	0.2926
PH	2009–2011 DS only	2012 DS	^A^ RKHS	0.2807
PH	2009–2011 DS only	2012 DS	^C^ BL	0.1886
PH	2009–2011 DS only	2012 DS	^D^ MLR	0.1132
PH	2009–2011 DS only	2012 DS	^B^ ped	0.2079
PH	2009–2011 all, 2012 DS	2012 WS	^D^ MLR	0.3174
PH	2009–2011 all, 2012 DS	2012 WS	^A^ RF	0.3000
PH	2009–2011 all, 2012 DS	2012 WS	^A^ RR-BLUP	0.2530
PH	2009–2011 all, 2012 DS	2012 WS	^A^ RKHS	0.2179
PH	2009–2011 all, 2012 DS	2012 WS	^C^ BL	0.0908
PH	2009–2011 all, 2012 DS	2012 WS	^B^ ped	0.1600

TP = Training population, all = both dry and wet seasons for each year, DS only = dry seasons only for each year. VP = validation population. Accuracy = correlation of the predicted GEBV and the phenotype in the validation population, where the training population included the validation season/year for individuals not in the validation fold. Statistical methods not connected by the same letter performed significantly different from each other across experiments by pairwise students t (α = .05).

**Grain yield**. The highest prediction accuracies for grain yield in both the 2012 dry and wet seasons were 0.31, when the training populations consisted of data from all four years (2009–2012), both seasons per year. The peak dry season accuracy was obtained when rrBLUP was used to build the model, and the peak wet season accuracy was obtained when RF was used (Table 2, S2 Table). In general, however, prediction accuracies did not significantly vary depending on the combination of years or seasons in the training population (α = 0.05). These results indicate that the most recent and complete years (2011, 2012) are also the most predictive, but that adding data from earlier years to the training population and utilizing both seasons of data (as opposed to using only the dry season to predict the dry season or only the wet seasons to predict the wet season) can marginally increase accuracy (Table 2, S2 Table). These results make sense given the strong correlations between the wet and dry seasons within the same year and the weak correlations between the earlier and later years for grain yield (S3 Fig.). The lower relative importance of the earlier year data could also be due to the large proportion of missing data in the earlier years.

The statistical method used to build the prediction model had a significant effect on accuracy. RR-BLUP, Random Forest, and RKHS all performed significantly better than pedigree alone. RR-BLUP and RF, specifically, outperformed pedigree prediction by an average of ∼8%. Similar results have been documented in CIMMYT wheat populations where genetic markers have been found to add 7.7%-35.7% to the accuracy of grain yield predictions over a pedigree-only model depending on the population and environment [52]. The modest gains in accuracy of using markers to predict breeding value in our rice population suggest that larger training populations may be necessary to better model the effects of Mendelian segregation on yield, in addition to effects due to family relationships [53].

Some of the marker models performed worse than pedigree prediction. Bayesian LASSO performed significantly worse than prediction based on pedigree alone, while MLR performed worst of all. It is worth noting that the GWAS for grain yield in this population (unlike the GWAS for flowering time or plant height) did not identify any large effect QTL [45], which could explain why choosing a subset of markers to predict GEBV performed so poorly relative to the genomic selection methods (Table 2, S2 Table).

**Flowering time**. The prediction accuracies for flowering time were higher than those for grain yield at 0.63 and 0.54 for the best performing experiments in the 2012 dry and wet seasons, respectively. For the dry season, the most predictive training population was composed of the 2009–2011 data, dry seasons only, while for the wet season, the best training population included all seasons from 2010–2011. The prediction accuracies for flowering time in the 2012 dry season were significantly higher than those for the 2012 wet season across statistical methods and experiments (p < 0.0001), but the differences in the performance of different training populations were not significant within a given validation population (Table 2, S3 Table).

Unlike for grain yield, the best accuracies for predicting flowering time for both seasons were obtained using MLR. In fact, MLR significantly outperformed all other statistical methods and was more accurate than pedigree alone by 22% and 33% for the dry and wet seasons, respectively (Table 2, S3 Table). The higher accuracies for prediction of flowering time relative to predictions for yield, and also of the dry season predictions over the wet season predictions, can be explained by the higher trait heritabilities for flowering time of the 2012 dry season relative to the 2012 wet season (Table 1), and by the strong correlation in the phenotype data for all years and seasons (S3 Fig.). The outstanding performance of MLR, on the other hand, is best explained by the genetic architecture of flowering time. Multiple large effect QTL have been cloned for flowering time [43,44], and the GWAS performed on this population identified a single very large effect QTL on chromosome 3 that explained more than 40% of the variation in flowering time [45]. These results are also consistent with results for prediction of heading date using MLR versus GS in wheat [51]. Of the genomic selection methods tested (MLR is a non-GS method), random forest performed the best by a significant margin, and was the next best method of predicting flowering time after MLR. This is worth noting as the random forest algorithm is also effective at capturing large-effect QTL [54].

Overall, these results suggest that the presence of large effect QTL for specific traits in rice could improve the prediction accuracy of those traits, although it remains to be seen whether genomic selection models will be the most practical means of obtaining those predictions. One promising avenue of research would be to model the large effect QTL as fixed effects using a genomic selection method such as rrBLUP [55].

**Plant height**. The accuracies for plant height were similar to those for grain yield, 0.34 for the dry season when the 2009–2011 dry seasons served as the training population, and 0.32 for the wet season when all seasons and years served as the training population (Table 2, S4 Table). These results further suggest that heritability has an important effect on accuracy. Both grain yield and plant height had similar heritabilities, and similar prediction accuracies (Tables 1, 2, S4 Table.)

For predicting plant height, however, MLR was sometimes the best-performing statistical method, as was the case for the most accurate wet season experiment, described above, but for other experiments, MLR was the worst-performing method, as for the best performing dry season experiment, described above. Due to the inconsistent performance of MLR, the prediction method with the best performance over all experiments was random forest (Table 2, S4 Table). Across all experiments, random forest outperformed pedigree prediction by an average of 13.3%, an improvement in the performance of marker based prediction relative to pedigree prediction that is squarely in between the improvements seen for grain yield and plant height (Table 2, S4 Table).

These results suggest that large marker effects help to make up the genetic architecture for plant height, but that plant height genetic architecture is more complicated than the genetic architecture of flowering time. This inference is borne out by the GWAS results for plant height—four large effect QTL were identified, explaining ∼74% of the total variation [45]. While these effects are large, they are not as dramatic as the one super-QTL found for flowering time on chromosome three, which may explain the difference in the performance of MLR for the two traits. As for flowering time, future research in predicting plant height could explore fitting these QTL in linear models as fixed effects.

### GS using marker subsets

**Fig 1:**
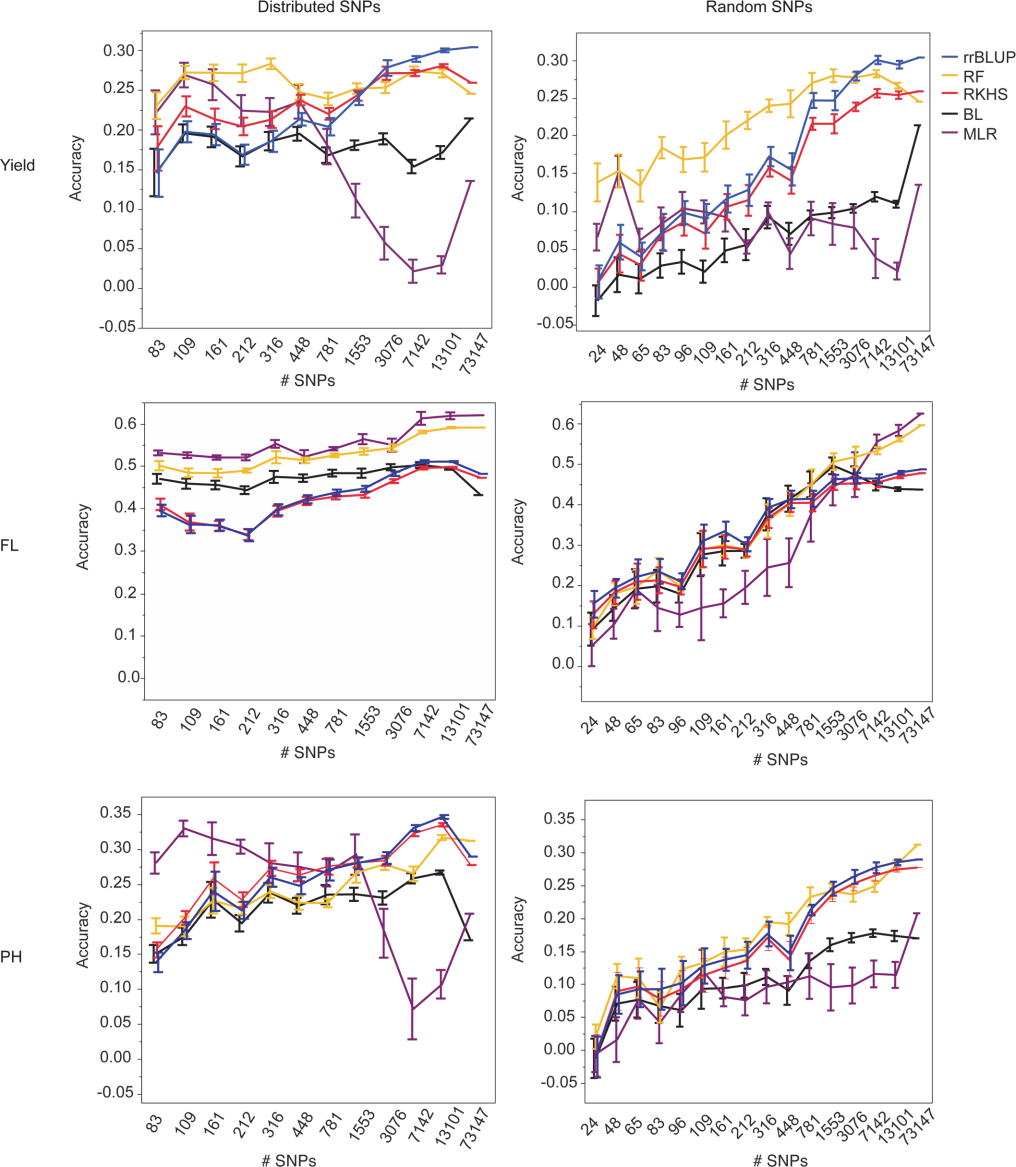
Mean accuracies of cross-validation for prediction of grain yield (Kg/ha) (top row), flowering time (days to 50% flowering) (middle row), and plant height (cm) (bottom row) in the 2012 dry season, using 10 selections of SNP subsets either distributed evenly throughout the genome (right column) or chosen at random (left column) and five different statistical methods, error bars constructed using 1 standard error from the mean. The training population consisted of data from years 2009–2011, both seasons per year.

In order to determine the necessary number of markers for performing GS in a rice population of this type, we selected differently sized SNP subsets from the 73,147 SNP set. The subsets were selected in two ways: 1. to be evenly distributed across the genome (see materials and methods for details) or 2. at random. Ten selections were made for each subset size and type (i.e. random versus distributed), and five-fold CV was performed using each selection in combination with all five marker based models (materials and methods). For each trait, cross validation was run for both validation populations, with years 2009–2011, both seasons per year, serving as the training population. (Fig. 1, S4 Fig., S5 Table, S6 Table).

For all three traits and both validation seasons, it is clear from the marker subset results that 73,147 markers is more than is necessary to capture the QTL segregating in this population. For almost all traits, there was no significant difference in the best-performing GS method for a given trait or validation season when 7,142 SNPs (approximately 1 SNP for every 0.2 cM) were used versus when 13,101 SNPs (1 SNP for every 0.1 cM) or the full 73,147 SNPs were used. This was true for the randomly chosen SNPs as well as for the evenly distributed SNPs, however the accuracy variances were higher for the randomly chosen SNPs, so it is our recommendation that SNPs be evenly distributed across the genome when possible (Fig. 1, S4 Fig., S5 Table, S6 Table). Although it is possible that the variation in the call rates and minor allele frequencies of the randomly selected SNPs also contributed to the larger variations in accuracy in the random SNP subsets, it is still thought that the position of the SNPs was the most important contributor to prediction accuracy.

For all three traits and both validation seasons, prediction accuracies dropped significantly faster with decreasing numbers of markers when the markers were chosen at random versus when they were evenly distributed throughout the genome. The drop-off in prediction accuracy when random selections of SNPs were used was particularly acute for flowering time and plant height and is attributable to the presence of large-effect QTL for these traits; As the number of randomly chosen SNPs decreases, the odds of capturing the effect of any one QTL also decreases. The prediction results for grain yield, by contrast, did not differ as dramatically between the randomly and evenly distributed subsets as did those for flowering time and plant height. These results suggest that the genetic architecture for grain yield is more in line with an infinitesimal model, i.e., that there are many small effect QTL throughout the genome, and are in agreement with the grain yield GWAS results [45]. It thus follows that the effect of choosing SNPs at random would not be as detrimental for grain yield as it is for flowering time or plant height when accuracy crucially depends on capturing specific regions that explain a high proportion of the phenotypic variance.

At fewer than 7,142 SNPs, accuracies began to decrease for most traits and statistical methods, although the extent to which accuracies decayed depended on the prediction method used, the trait, and the validation season. For grain yield in the 2012 dry season, for example, there was no significant difference in the performance of rrBLUP at any marker set > = 3076 markers. For random forest, however, there was no significant difference in prediction accuracy all the way down to sets of markers > = 316 (random or distributed). While it would not be advisable to use such a small number of markers, as the smaller the number of markers, the larger the variation in prediction accuracy, these results do suggest that for grain yield, at least, random forest works better with smaller numbers of markers than does rrBLUP. The results for plant height were very similar to those for grain yield. For flowering time, when SNPs were evenly distributed, variances in accuracy were very small, again, most likely as a result of the super-QTL on chromosome three. These very small variances meant that for both MLR and random forest, accuracies were significantly lower for fewer than 7142 SNPs (distributed) or 1553 SNPs (random).

Taken collectively, these results suggest that using ∼1 SNP every 0.2 cM (∼6–7K SNPs), could be ideal for performing genomic selection in inbred rice breeding populations like the one at IRRI. Opportunely, two Infinium 6K SNP fixed arrays have recently been developed for use within specific rice breeding/research programs [56]. Fixed arrays have established advantages in rice, including robust allele calling, cost-effectiveness per data point, and speed of genotyping turn-around [56]. 6–12K fixed arrays could thus prove to be the most affordable and efficient means of genotyping for GS in rice, especially for smaller breeding programs with less genotyping informatics expertise. The best strategy, however, will likely be to have multiple genotyping platforms available and the flexibility to switch between them as needed. Genotyping turn-around time is ultimately key for GS because genotypes must be available in time for selections and the next generation of crossing. It should be noted that depending on the platform, genotyping individuals with more markers than is necessary could be detrimental to breeding progress if it overloads the bioinformatics and computational capacities of a breeding program.

### A GWAS-GS joint venture

The matrix of genotypes and phenotypes on a breeding population provides the opportunity to perform GWAS in addition to testing any GS models that are available. This paper describes the GS-side of a joint GS-GWAS project on a single rice breeding population, and is the first study to suggest that GWAS on a set of breeding lines might provide information about both the genetic architecture of the traits-of-interest and the population structure of the breeding materials. Specifically, our results on performing GS for grain yield, plant height, and flowering time demonstrate that performing GWAS using the inputs to GS can reveal the presence of large-effect QTL segregating in a breeding population, which can then be modeled accurately using GS.

### The future of GS in rice

Our results are promising for the implementation of GS in rice improvement. For all traits tested, GS outperformed prediction based on pedigree alone with the use of a reasonable number of markers (∼7000) suggesting that genomic selection is accessible for moderately-resourced public programs with minimal bioinformatics capacities. For yield, which appears to be controlled by many genes of small effect [45], RR-BLUP was the most computationally efficient of the best performing statistical methods. For plant height and flowering time, however, the highest accuracies were obtained using random forest and/or MLR, which suggests the presence of both large and small effect QTL for these traits, a hypothesis that is also supported by the GWAS results [45].

Currently, the most commonly used methods of rice improvement are pedigree breeding and traditional marker assisted selection, which mainly track large effect QTL. Our results suggest that genomic selection will make it possible for the first time to track, accumulate, and select for small effect QTL using genetic markers in addition to large effect QTL. One promising strategy is to build GS models in which large effect QTL are fit as fixed effects to capture the variance of large-effect QTL along with small effect QTLs located throughout the genome [55]. Future experiments in rice genomic selection should focus on building these models.

**Fig 2:**
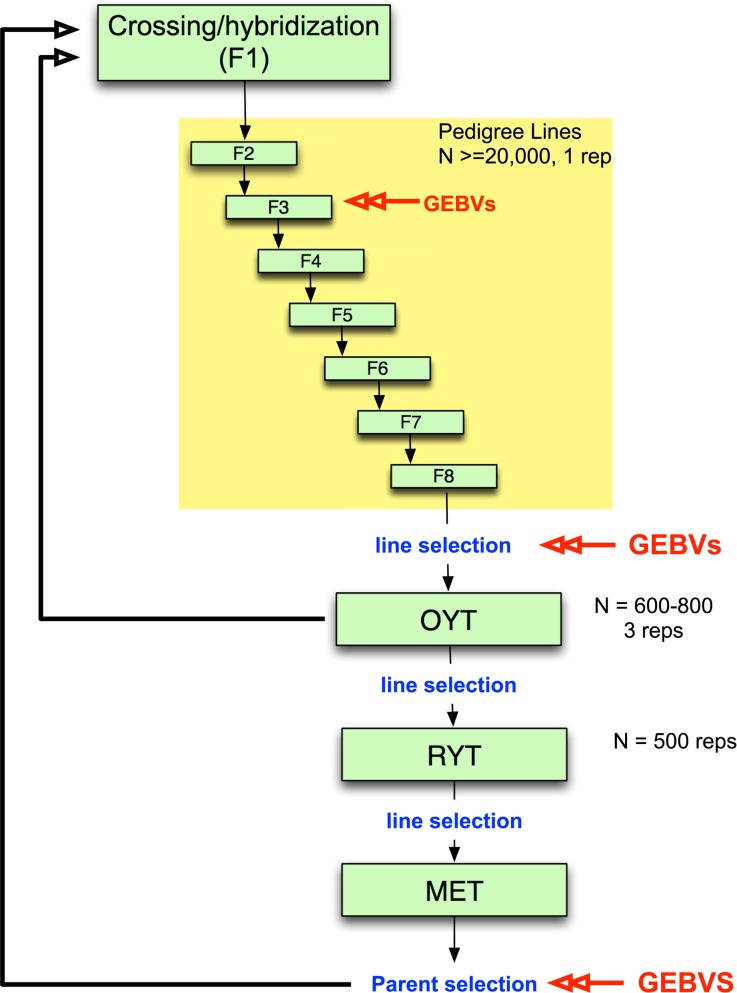
Example of irrigated rice breeding pipeline that incorporates genomic selection. Parents are selected and crossed to create an F1 population. ∼20,000 F1 lines are fixed over 7–8 generations with selection of families for heritable traits with ∼25% of the pedigree lines eventually selected for entry into the observational yield trial (OYT). GEBVs can be used at two or more generations during fixation as resources permit to perform selection. Here we propose using GEBVs at the F3 and F6 generations. GEBVs are also used to select the fixed lines from the F8 to advance to the OYT. The top lines advanced to the OYT based on GEBV are cycled back into the crossing block in order to continue to improve the population. From the OYT, the best performing lines based on phenotype are advanced to the replicated yield trials (RYT), and the best performing lines from the RYT are advanced to the multi-environment trials (MET). Lines from the MET are then selected based on GEBV as parents for the next generation of recurrent selection. Models are built at each stage in which GEBVs are used for selection based on a subset of the lines in the population (∼300 individuals representing different families) that are both genotyped and phenotyped to form the training set. The rest of the individuals in the population are genotyped only in order to calculate GEBVs.

While genomic selection has yet to be integrated into applied breeding programs in rice as it has in maize and wheat, it would be feasible to undertake small pilot programs within specific rice breeding programs, especially for irrigated rice where growing environments are generally more uniform. Such pilot programs are needed, in particular, to determine when and how to incorporate genomic selection into existing breeding programs. An example of an irrigated rice breeding pipeline that incorporates genomic selection is presented in Fig. 2. Parents are selected and crossed and the resulting F1 progeny fixed over seven generations with selection of families for heritable traits. Traditionally, selection during pedigree line fixation would be based only on phenotype. Here, we propose incorporating selection based on GEBV at least once during fixation, as resources allow. Early generation GEBV-based selection would help to avoid eliminating families that carry beneficial rare or recessive alleles and would increase the proportion of top performers that are advanced to the observational yield trials (OYT). Late-generation selection based on GEBVs could be used to select fixed lines to advance to the OYT. The top lines advanced to the OYT based on GEBV could be used simultaneously as parents of the next generation of breeding (Fig. 2).

From the OYT, the best performing lines could be identified and advanced to the replicated yield trials (RYT) by a combination of phenotypic and genomic selection. Phenotypic selection by the breeder has the potential to compensate for the fact that the GS model is always a generation or more behind the current breeding population. This means that any favorable new GxG interactions will not be captured by the model and cannot be selected by GEBV alone. In species where the majority of the genetic variation under selection is controlled by many additive, small effect loci, this should not be a problem. However, in rice and other inbreeding crops, the genetic architecture of many important agronomic traits contains important non-additive features and transgressive variation is common [41,43,57,58,59,60]. The selected lines from the RYT are subsequently advanced to the multi-environment trials (MET) where the GEBVs can be used to select parents for the next generation of hybridization. In order to build or update the genomic selection model at any stage of selection, a training set consisting of a fraction of the breeding population (∼300 individuals) representing different families would need to be both phenotyped and genotyped. The rest of the lines would be genotyped only to calculate the GEBVs (Fig. 2).

The above genomic selection models would ideally account for multiple environments and GxE interactions, however current programs such as the one at IRRI and many other national breeding institutes do not make use of multi-environment data until very late stages of the breeding process, after the population has already been reduced to a manageable number of lines. Thus, even GS models that do not account for multiple environments, like those presented here, are of use to plant breeders and have the potential to improve breeding outcomes. The data from the Multi-environment trials on the IRRI breeding lines used in this experiment is currently being accumulated and vetted and will be a subject for future GS research.

In order to fully exploit the benefits of GS, however, new rice breeding schemes will need to be implemented to further reduce the breeding cycle and increase genetic gain. Heffner et al. (2010) proposed a GS scheme for winter wheat using rapid generation advance (RGA) to generate F5 lines, and multi-location field trials to test F5-derived material, which was further used to train the GS model [13]. A similar scheme should also be effective for rice and a modified scheme is currently being implemented at IRRI within the irrigated breeding program.

By using the genotype and phenotype inputs from pilot programs for both GS and GWAS, the accuracy of GS models could be improved while, at the same time, helping to answer basic biological questions about the genes underlying agronomic traits of interest. Ultimately, in order for genomic selection to be of practical use, it must be possible to select lines with combinations of phenotypes that are routinely measured by breeders, such as disease and insect resistance and grain quality. GEBVs could be used to select for traits that are either difficult or expensive to phenotype or are late in development (e.g. panicle or post-harvest traits), while phenotypic selection, such as in the OYT and RYT in Fig. 2, could be used for other important variety parameters. The use of multi-variate GS models or selection indices as GS phenotypes are other potential solutions to this problem, but both require additional research and computational/statistical inputs to implement.

In practice, determination of whether GS can cost-effectively increase genetic gains relative to utilizing pedigree data alone or simply phenotyping more lines requires a careful consideration of the relative cost of phenotyping compared to genotyping plus line development [61]. For GS to provide increased genetic gain in a pedigree breeding program, the prediction approach must either increase accuracy relative to phenotyping or permit a substantial increase in selection intensity. It is possible to increase selection intensity through the use of rapid generation advance, as mentioned above, but the selection intensity increase must be very large because the response of genetic gains to increasing selection is logarithmic rather than linear.

In this study, GEBV accuracy for yield averaged about 0.3 for the most effective prediction methods (Table 2). The corresponding accuracy for phenotypic selection is the square root of heritability, or about 0.55 for evaluation in a single three-replicate trial (Table 1). An accuracy of 0.3 corresponds to a heritability for yield evaluation of 0.1, which is roughly the accuracy achievable by screening for yield in a single unreplicated irrigated rice trial at IRRI (e.g. Bernier *et al*., 2007). Currently, the cost of phenotyping a single rice plot for yield and genotyping via GBS is roughly equivalent ($20-$30), so there is no clear advantage for GS over simply phenotyping more materials in unreplicated trials. However, genotyping costs are likely to continue to drop, whereas phenotyping costs are generally steady or rising. Furthermore, continued refinement of GS models by incorporating fixed effects and accumulation of high quality data over years and environments is expected to increase GEBV accuracy. As a result, we predict that in the near future, GS will become a cost-effective means of performing line selection in rice.

## Materials and Methods

### Plant material

369 elite breeding lines were selected for genotyping from the International Rice Research Institute (IRRI) irrigated rice breeding program based on the planned inclusion of the lines in the 2011 Multi-Environment Testing Program and presence in the 2011 and 2012 Replicated Yield Trials (RYT) at IRRI (Los Baños). Approximately half of the lines were also included in the 2009–2010 RYTs at IRRI (S1 Table). The other lines were promoted from the observational yield trial (OYT) to the RYT in 2011.

### Phenotyping

Phenotypes for the replicated yield trials (RYT) were used for all the experiments and curated from the IRRI database for years 2009–2012, including wet and dry seasons each year. All of the RYT breeding lines, of which our selected 369 lines are a subset, were grown in a randomized complete block design with three replicates in the same field location at IRRI every season and year. The following data were curated for each year, with the exception that plant height data was not available for the 2009 wet season:

- plant height: the actual measurement in cm from soil surface to tip of tallest panicle (awns excluded)
- flowering time: days to when 50% of flowers were visible in whole plot
- maturity date: days to when 85% of grains on panicle were mature
- number of effective tiller or panicle per plant: count of the number of panicles on each plant
- lodging score: percent of plants that lodged
- grain yield (kg/ha): grain yield from a representative plot was harvested and weighed, from this sample the grain yield per hectare was calculated from an inner harvested area of the plot excluding border rows
- rep: replication number of observation

The plant height, flowering time, and grain yield phenotypes were selected for prediction using the genomic selection models.

### Genotyping

**DNA extraction**. Young leaf tissue was collected from each of the 369 breeding lines from plants grown in Gutterman Greenhouse in Ithaca, NY. DNA was extracted using the Qiagen 96-plex DNeasy kit as per the Qiagen fresh leaf tissue 96-plex protocol (www.qiagen.com/HB/DNeasy96Plant).

**Library preparation**. 384-plex genotyping-by-sequencing (GBS) libraries were prepared using the protocol by Elshire *et al*. 2011 [62], as described previously in Spindel and Wright *et al* 2013 [63].

**GBS data analysis**. SNPs were discovered and called from the raw 384-plex GBS data using the TASSEL3.0 GBS pipeline with physical alignment to the MSU version 6.0 Nipponbare rice reference genome using Bowtie2, as described in Spindel and Wright *et al* 2013 [47,63,64] (S5 Fig.). The IRRI breeding materials genotyped here are a collection of multi-parent related and unrelated inbred lines, so the GBS-PLAID algorithm for imputation, which was developed specifically for imputation of biparental rice mapping populations, was not useful [63]. Imputation of missing data was instead performed using the TASSEL3.0 FastImputationBitFixedWindow plugin with default settings [48]. The algorithm works by dividing the entire SNP dataset into small SNP windows, then identifying the most similar inbred line within each window to fill the missing data. The algorithm takes advantage of small IBD regions shared between pairs of inbred lines in the collection; if the window from the closest neighbor has more than 5% difference from the line being imputed, the data point is left as missing [48]. The imputation error rate using this algorithm was estimated for each chromosome in our dataset by masking a fraction of the un-imputed allele calls and comparing the imputed and actual calls. The average imputation error rate across the twelve rice chromosomes was estimated in this way to be less than 1%.

SNPs that still had 10% or more data missing after imputation (or call rates of < 90%) were removed from the dataset along with all monomorphic SNPs, for a total SNP set of 73,147 SNPs. After the SNP filtering described above, individuals with more than 60% missing data were dropped from the dataset, which resulted in the removal of six individuals that failed sequencing for the total of 363 genotyped lines used throughout the study (S5 Fig.).

The final dataset was then transformed from nucleotide genotype coding (i.e., 'A', 'C', 'T', 'G') to numeric coding (1, 0, -1 for class I homozygotes, heterozygotes, and class II homozygotes, respectively) to facilitate statistical analysis. The minimal remaining missing data were filled using the numeric genotype means of each line in order to perform PCA and genomic selection modeling (S5 Fig.).

### Subpopulation and family structure analysis and cross-validation fold design

The majority of the 363 lines were characterized *a priori* from pedigree records to belong to the *indica* or *indica-admixed* subpopulation groups. In order to identify outlier individuals belonging to the *japonica* or *japonica-admixed* groups, principle components analysis (PCA) was performed in R (version 3.0.1) using the imputed 73,147 SNPs, with remaining missing data filled using the line means. The first principal component of high density SNP data in rice can separate the *indica* and *japonica* subgroups [30], so by plotting the first four principal components using JMP Pro 10, 13 *japonica* outliers were identified as a tight cluster that was pulled apart from the rest of the 350 lines (S1A Fig.). These 13 lines were removed from the dataset, and a second PCA was performed using the same methodology as the first to identify any admixed outliers, i.e, outlier lines containing greater percentages of *japonica* derived SNPs. By plotting the first four principal components of the second PCA, another 18 lines were judged on a visual basis to be outliers and removed from the dataset, leaving a total of 332 lines to be used for the cross-validation experiments (S1B Fig.). A third PCA was performed using the remaining 332 to confirm that there were no additional subpopulation outliers.

It was also known from studying the breeding program pedigrees that differing degrees of family relatedness existed within the remaining 332 lines, including half sibs, full sibs, parents and offspring, and unrelated lines. The presence of highly related individuals in the dataset could have the effect of artificially inflating prediction accuracy if the most closely related individuals are randomly assigned to different folds, and one of those folds is then used as training, while the other is used as testing. Or, in other words, the training fold could end up as unusually predictive of the testing fold if, for example, a pair of full sibs is split across training and testing folds. To control for this possibility when designing our folds, we performed a partitioning around k-medoids analysis (pamk) using the R fpc package (function pamk) with the 73,147 SNPs. k values from 2 to 332 were tested to determine the most statistically probable k-value by average silhouette width (S2 Fig.). The largest average silhouette width was found to occur at k = 87 (S2A Fig.). Individuals found within same cluster of 87 were then assigned to the same fold, making it impossible for the most closely related individuals to be split across training and testing folds. Full clusters were assigned to one of five folds randomly, controlling only for cluster size in order to produce three folds of 66 individuals and two folds of 67 individuals. A similar procedure was used by Ly et al., 2013 [26].

### Cross-validation experimental design

For each cross validation experiment, one of the five folds served as the validation fold, and the other four folds served as the training folds. The process was repeated five times so that each fold served once as the validation fold, resulting in predicted GEBV values for all individuals. Accuracy was assessed as the mean Pearson Correlation of the predicted GEBV and observed phenotype in the validation population.

The cross validation experiments shown in Table 3 were performed in order to test all logical combinations of years and seasons in the training and validation populations. Note that a year's wet season was never used to predict the same year's dry season because in Southeast Asia, the dry season arrives first chronologically. We did, however, predict the 2012 wet season both with and without the 2012 dry season present in the training population. We tested scenarios in which both seasons per year were included in the training population as well as scenarios where only the data from the seasons matching the validation population were included in the training data (e.g., using only the wet season data to predict the wet season). We also sought to test scenarios using only more recent year data in the training population (e.g. only 2011, or 2010–2011) and scenarios using more historical year data in the training population (e.g. 2009–2011) (Table 3).

**Table 3:** CV experiments.

Experiment Numbers		
	VP	
TP	2012DS	2012WS
2009–2011 AS	1	3
2009–2011 AS, 2012DS		2
2009–2011 DS	4	
2009–2011 WS		5
2011 AS	6	7
2011 AS, 2012 DS		8
2010–2011 AS	9	10
2010–2011 AS, 2012 DS		11
2010 WS, 2011 AS	12	

*AS = all seasons, DS = dry season only, WS = wet season only

### Inclusion of validation population year/season in training population

Cross validation experiment 1 (CV1) accuracies were calculated for all experiments with the validation year/season included in the training population, excluding individuals in the validation fold. Including the validation year/season in the training population can bias accuracies upwards by confounding GxE and line effects, however, so in order to obtain an estimate of this bias, we also performed cross validation experiments 2 and 3 (CV2, CV3) for CV permutations 1–5, see above table. For CV2, we excluded the validation year/season from the training population. These results are not directly comparable to those in which the training population contained the validation year/season (CV1), however, because the training population for CV2 is smaller than was used for CV1 and training population size can have an important effect on prediction accuracy. For this reason, we performed CV3, in which we included the validation year/season in the training population, but removed the equivalent seasons from 2011, e.g., for the first cross-validation permutation in the above table, CV2 would not include the 2012 dry season in the training population, and CV3 *would* include the 2012 dry season but would *not* include the 2011 dry season. Thus, the estimate of bias can be calculated for a given CV permutation experiment as CV3 accuracy minus the CV2 accuracy [26]. The bias was only estimated for the first five CV permutations because the bias estimates turned out to be small and similar to each other for all five CV permutations.

### Calculation of adjusted phenotypes for validation folds and correlation analyses

For all three traits, multiple years, seasons, and replicate yield entries existed along with the previously described covariates for all 332 individuals. In order to build genomic selection models, it was necessary to convert these raw yields into a single, adjusted yield for each individual. Adjusted yields, plant heights, or days to flowering were calculated for each year/season combination by fitting an initial linear model of the observations *y*, by line ID (GHID) *x*_*1*,_ and phenotype covariates described above (e.g. lodging) *x*_*2*…*n*_ for the given Year x Season in JMP. Non-significant covariates as determined by an F-test (α > = 0.05) or covariates that resulted in singularities were removed, and the model re-fit. When all covariates included in the model were statistically significant, the least squares mean yield for each line ID was exported as the adjusted yield. Missing phenotype data were coded as null data for the above analysis, or, in other words, no imputation or numeric filling of phenotypic values was performed.

The least square means for each year and season were also used to calculate a correlation matrix for each trait (S3 Fig.).

### Calculation of adjusted phenotypes for training folds

For each experiment, adjusted yields were calculated for each of the five training folds separately by fitting a linear model for each training fold as described above with the difference that data from all years and seasons for a particular CV experiment was including in the x matrices for all lines not in the validation fold. Year, season, and a year x season interaction were also included as covariates in the model, and subject to the same significance requirements as the other model covariates.

### Genomic selection and pedigree modeling

Six statistical methods were used for each experiment, including four genomic selection methods: RR-BLUP, Bayesian LASSO (BL), Reproducing Kernel Hilbert Spaces (RKHS), and Random Forest (RF), and two non-genomic selection methods: Multiple Linear Regression (MLR) and Pedigree-BLUP (PED). The four genomic selection methods were chosen based on their demonstrated success in accurately predicting GEBV in variety of crops and because they represent the different types of statistical methodologies used to build GS models, i.e., Linear parametric methods (RR-BLUP, BL), non-linear semi-parametric methods (RKHS), non-linear, non-parametric methods (RF), as well as Frequentist methods (RR-BLUP, RKHS), Bayesian methods (BL), and machine learning methods (RF) [19,23,49,50,65,66,67]. For an overview of the methods, see Lorenz *et al*., 2011[8].

Multiple linear regression using a subset of markers derived from single marker regressions (MLR), another linear, parametric statistical method was the fifth statistical method tested to predict breeding value, and served as our used as a non-GS marker-based prediction control. For each fold, single marker regression was run for all markers and p-values determined for each marker by f-test. Note that this is the statistical equivalent of a crude GWAS. Linear models were then tested using 1 through the first 100 most significant markers, and the model with the best fit was returned. The returned model was then used to calculate the accuracy for the given fold. For the marker subset experiments where the number of markers (p) was less than 100, models were tested using 1 through p markers. MLR has been shown to be effective for agronomic traits with very simple genetic architectures, but is otherwise not expected to perform well [51].

Prediction based on pedigree alone was the sixth statistical method and was performed in order to determine if a.) the fold design method properly controlled for family structure within the dataset, and b.) if GS could outperform prediction based on pedigree alone [52].

All statistical modeling was done in R. For the pedigree models an A-matrix was calculated using a three-generation pedigree file for all individuals in the training and validation populations using a custom R function. The models themselves were calculated using package rrBLUP (function kin.BLUP). RR-BLUP models were also calculated using package rrBLUP (function kinship.BLUP). RKHS models were calculated using kinship.BLUP, K.method = "GAUSS", modified so that parameter theta was always equal to 2.5, as per guidelines in the BGLR package documentation [68]. Random Forest was performed using package randomForest (function randomForest). Bayesian LASSO was performed using package BLR (function BLR).

Narrow sense heritabilities were calculated for each trait on a per line basis using the rrBLUP package, function mixed.solve, with the least square means for the complete validation populations used as input. The narrow sense heritabilities were calculated as the additive genetic variance divided by the total phenotypic variance. The set of 73,147 SNPs was used for all experiments with the exception of the marker subset experiments described below.

The cross-validation results were analyzed using ANOVA and pairwise student's t to determine:

a significant difference in the accuracy of prediction of the two validation populations across statistical methods, i.e., where *y*_*i*_ (accuracy) = *μ* + *x*_*ij*_ *β*_*j*_ + *ε*_*ij*_, and *i* is one RYT experiment and stat method for validation population *j* (e.g. *x*_*i*_ *=* CV experiment 1 for method RR-BLUP and *j* = validation population 2012 DS).
b significant difference in the performance of the six statistical methods across the different experiments, i.e., where *y*_*i*_ (accuracy) = *μ* + *x*_*ij*_ *β*_*j*_ + *ε*_*ij*_, and *i* is one RYT experiment for stat method *j* (e.g. *x*_*i*_ = CV experiment 1 and *j* = RR-BLUP).
c significant difference in the performance of each experiment across statistical methods, after excluding the three worst-performing statistical methods (Bayesian LASSO, MLR, and pedigree only), i.e., where *y*_*i*_ (accuracy) = *μ* + *x*_*ij*_ *β*_*j*_ + *ε*_*ij*,_ and *i* is one statistical method for RYT experiment *j* (e.g. *x*_*i*_ = RR-BLUP and j = CV experiment 1) (Table 1, S2–S4 Tables).

### Cross validation using random and distributed SNP subsets

**Distributed**. To select subsets of SNPs that were evenly distributed across the genome, 11 bin parameters were selected: 25Kb (0.1 cM), 50 Kb (0.2 cM), 120 Kb (.5 cM), 240Kb (1 cM), 480 Kb (2 cM), 840 Kb (3.5 cM), 1200 Kb (5 cM), 1800 Kb (7.5 cM), 2400 Kb (10 cM), 3600 Kb (15 cM), 4800 Kb (20 cM). For each bin parameter, all SNPs in the 73,147 SNP set were placed into bins according to the bin parameter. To select subsets of SNPs for a given bin size, the SNPs in each bin were sorted first by minor allele frequency, largest to smallest, and then by call rate, largest to smallest. Ten selections of SNPs were made for each bin size—the first subset consisted of the top ranked SNP in each bin, i.e., the SNP with the highest MAF and call rate, the second subset consisted of the second ranked SNP in each bin, and so on for the top ten SNPs in each bin. If a bin had fewer than ten SNPs, then the top SNP in each bin was chosen for all ten selections.

Each subset was then used as the genotype matrix to perform five-fold cross-validation using the same folds as for the original RYT cross validation experiments. The RYT 2012 wet season and the RYT 2012 dry season served as the validation populations and RYT years 2009–2011, all seasons, served as the training population. The five marker-dependent statistical methods tested previously were used once more: RR-BLUP, RKHS, Random Forest, Bayesian LASSO, and MLR. Accuracy was calculated for each of the ten selections (for each bin parameter) as previously. A mean accuracy, standard deviation, and standard error for each bin parameter were also calculated by averaging the cross-validation results of the 10 selections for each bin parameter (S5 Table).

The average accuracies with standard error as the error bars were plotted versus the number of SNPs in each subset (as determined by the bin size parameter) using JMP (Figs. 1, S4). The results for full 73,147 SNP set were included on these plots as a reference, although these accuracies are not averages. ANOVA and pairwise students were used to test for significant difference in the performance of the five statistical methods across the different bin parameter sizes, and for significant differences in the performance of the various bin parameter sizes (and thus total SNP number) across the five statistical methods (S5–S6 Tables).

**Random**. Ten random selections of SNPs were chosen from the 73,147 SNP set for 15 subset sizes: 24, 48, 65, 83, 96, 109, 161, 212, 316, 448, 781, 1553, 3076, 7142, 13101 using a pseudo-random numbers generator. Subset sizes 83, 109, 161, 212, 316, 448, 781, 1553, 3076, 7142, and 13101 were chosen to match the number of SNPs in the distributed SNP subsets described above. The additional SNP subset sizes were included to improve resolution.

Cross validation experiments and analysis were performed for the random subsets as described above for the distributed subsets (Fig. 1, S4 Fig., S6 Table).

## Supporting Information

S1 Fig Plots of the first four principle components of selected elite breeding lines using 73,147 SNPs. (**A**) Initial principle components analysis (PCA) using 363 lines to identify 13 *japonica* outliers (purple). (**B**) PCA on remaining 350 lines after removing 13 outliers identified in A. An additional 18 outliers (purple) were subsequently identified and excluded.
(EPS)

S2 Fig Partitioning around K-medoids analysis of remaining 332 genotyped lines after removal of subpopulation outliers. Plot shows the number of clusters versus the average silhouette distance (asw). Maximum asw occurs at k = 87, suggesting this is the most statistically probable number of clusters within the 332 lines.
(EPS)

S3 Fig Cell plots showing the least square mean correlations for grain yield (top), flowering time (middle) and plant height (bottom) for each year and season. (EPS)

S4 Fig Mean accuracies of cross-validation for prediction of grain yield (Kg/ha) (top row), flowering time (days to 50% flowering) (middle row), and plant height (cm) (bottom row) in the 2012 wet season, using 10 selections of SNP subsets either distributed evenly throughout the genome (right column) or chosen at random (left column) and five different statistical methods, error bars constructed using 1 standard error from the mean. The training population consisted of data from years 2009–2011, both seasons per year.
(EPS)

S5 Fig Diagram of genotyping process. 384-plex GBS was used to discover and call SNPs on 369 elite inbred rice lines from the IRRI irrigated rice breeding program. SNPs were discovered and called from the raw GBS data using TASSEL3 with physical alignment to the MSU version 6 Nipponbare rice reference genome using Bowtie2 (yellow boxes). Imputation of missing data was then performed using the TASSEL3 fastimputationbitfixedwindow plugin as it was well suited to this collection of multi-parent inbred lines in which many regions across the lines were identical by descent (methods). After imputation, custom python scripts (green boxes) were used to remove SNPs with call rates < 90%, remove monomorphic SNPs, drop individuals with more than 60% missing data, and finally, convert the ACTG nucleotide calls to numeric coding (i.e., homozygote class I = 1, homozygote class II = -1, heterozygote = 0). After genotypes were converted to numeric format, remaining missing genotype values were filled using the numeric line mean.
(EPS)

S1 Table Summary of missing data for the remaining 332 genotyped lines (after outlier removal) by year and season. (DOCX)

S2 Table Complete GS cross-validation results for grain yield in the 2012 dry season (2012 DS) and the 2012 wet season (2012 WS) using 73,147 SNPs. TP = Training population, all = both dry and wet seasons for each year, DS only = dry seasons only for each year, WS only = wet seasons only for each year. VP = validation population. Accuracy = correlation of the predicted GEBV and the phenotype in the validation population, where the training population included the validation season/year for individuals not in the validation fold. Accuracy exp type 2 = correlation of the predicted GEBV and the phenotype in the validation population, where the validation year/season is not included in the training population. Accuracy exp type 3 = correlation of the predicted GEBV and the phenotype in the validation population, where the validation year/season is included in the training population, but the equivalent set of data is removed from year 2011. Bias estimate = Accuracy exp type 3 minus Accuracy exp type 2. Statistical methods not connected by the same letter performed significantly different from each other across experiments by pairwise students t (α = .05).
(XLSX)

S3 Table Complete GS cross-validation results for flowering time in the 2012 dry season (2012 DS) and the 2012 wet season (2012 WS) using 73,147 SNPs. TP = Training population, all = both dry and wet seasons for each year, DS only = dry seasons only for each year, WS only = wet seasons only for each year. VP = validation population. Accuracy = correlation of the predicted GEBV and the phenotype in the validation population, where the training population included the validation season/year for individuals not in the validation fold. Accuracy exp type 2 = correlation of the predicted GEBV and the phenotype in the validation population, where the validation year/season is not included in the training population. Accuracy exp type 3 = correlation of the predicted GEBV and the phenotype in the validation population, where the validation year/season is included in the training population, but the equivalent set of data is removed from year 2011. Bias estimate = Accuracy exp type 3 minus Accuracy exp type 2. Statistical methods not connected by the same letter performed significantly different from each other across experiments by pairwise students t (α = .05).
(XLSX)

S4 Table Complete GS cross-validation results for plant height in the 2012 dry season (2012 DS) and the 2012 wet season (2012 WS) using 73,147 SNPs. TP = Training population, all = both dry and wet seasons for each year, DS only = dry seasons only for each year, WS only = wet seasons only for each year. VP = validation population. Accuracy = correlation of the predicted GEBV and the phenotype in the validation population, where the training population included the validation season/year for individuals not in the validation fold. Accuracy exp type 2 = correlation of the predicted GEBV and the phenotype in the validation population, where the validation year/season is not included in the training population. Accuracy exp type 3 = correlation of the predicted GEBV and the phenotype in the validation population, where the validation year/season is included in the training population, but the equivalent set of data is removed from year 2011. Bias estimate = Accuracy exp type 3 minus Accuracy exp type 2. Statistical methods not connected by the same letter performed significantly different from each other across experiments by pairwise students t (α = .05).
(XLSX)

S5 Table Complete GS cross-validation results using distributed subsets of SNPs for prediction of grain yield, flowering time, and plant height in the 2012 dry and wet seasons. Distribution of SNPs was based on a bin parameter, i.e., the genome was divided into bins of a certain size, then ten subsets of SNPs were selected by choosing the SNPs in each bin with the largest minor allele frequencies and highest call rates. Training population = RYT 2009–2011 all. Cells in the SNP number column (within a particular trait and validation season) not connected by the same letter indicate a significant difference in the mean CV accuracy for this bin size for a given statistical method by pairwise student's t, α = 0.05 (each validation population and statistical method were run separately). Cells in the stat method column not connected by the same letter (within a particular trait and validation season) indicate a significant difference in the performance of this statistical method (versus the other four statistical methods) for a given bin size by pairwise student's t, α = 0.05 (each validation population and SNP number were run separately). All traits and validation seasons were also run separately.
(XLSX)

S6 Table Complete GS cross-validation results using random subsets of SNPs for prediction of grain yield, flowering time, and plant height in the 2012 dry and wet seasons. For each number of random SNPs, ten random subsets of the 73,147 SNP set were chosen. Training population = RYT 2009–2011 all. Cells in the SNP number column (within a particular trait and validation season) not connected by the same letter indicate a significant difference in the mean CV accuracy for this bin size for a given statistical method by pairwise student's t, α = 0.05 (each validation population and statistical method were run separately). Cells in the stat method column not connected by the same letter (within a particular trait and validation season) indicate a significant difference in the performance of this statistical method (versus the other four statistical methods) for a given bin size by pairwise student's t, α = 0.05 (each validation population and SNP number were run separately). All traits and validation seasons were also run separately.
(XLSX)
