# Correction: Genomic Selection and Association Mapping in Rice (*Oryza sativa*): Effect of Trait Genetic Architecture, Training Population Composition, Marker Number and Statistical Model on Accuracy of Rice Genomic Selection in Elite, Tropical Rice Breeding Lines

**DOI:** 10.1371/journal.pgen.1005350

**Published:** 2015-06-30

**Authors:** Jennifer Spindel, Hasina Begum, Deniz Akdemir, Parminder Virk, Bertrand Collard, Edilberto Redoña, Gary Atlin, Jean-Luc Jannink, Susan R. McCouch

The legend for Fig 1 is incorrect. Please read the corrected legend below:

**Fig 1:**
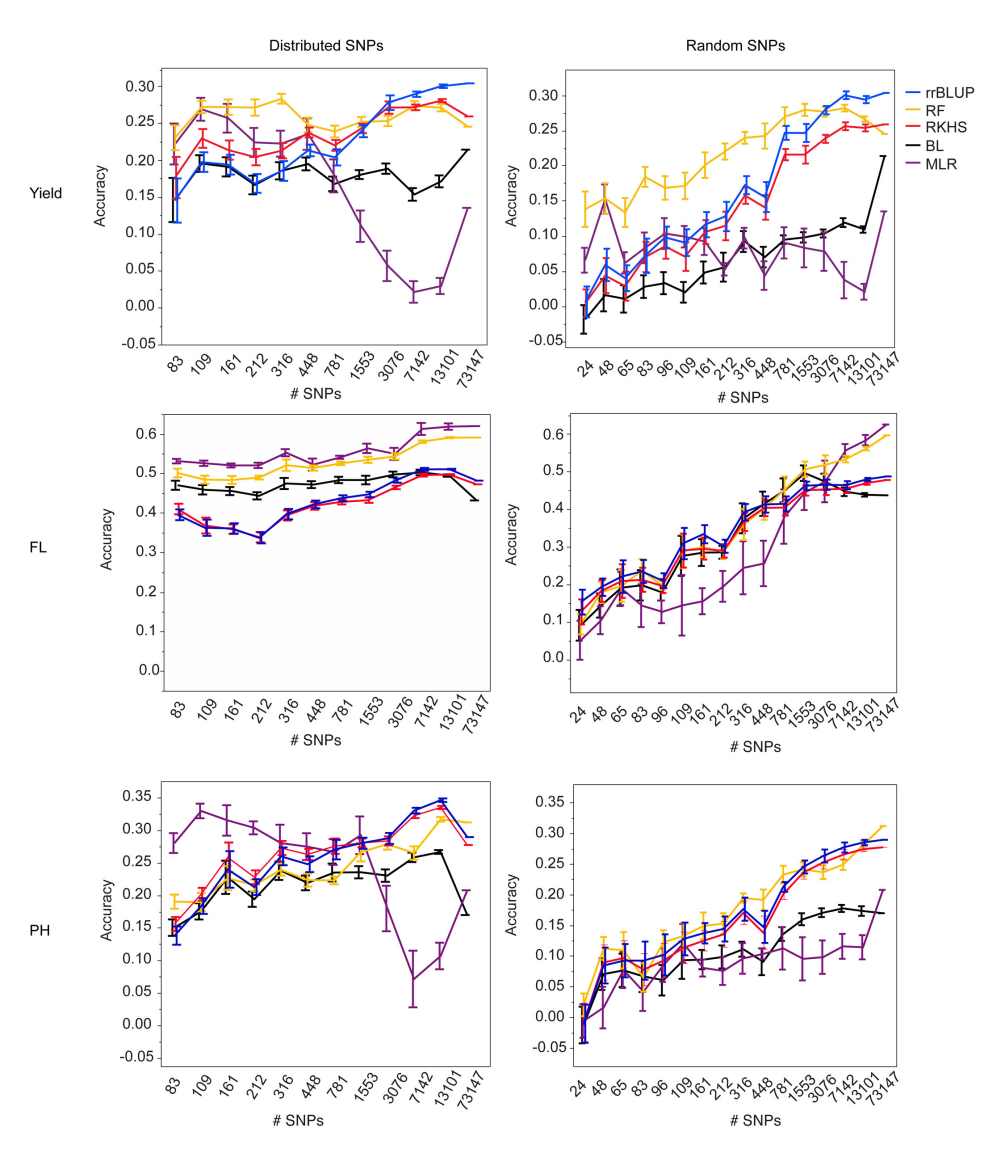
Mean accuracies of cross-validation for prediction of grain yield (Kg/ha) (top row), flowering time (days to 50% flowering) (middle row), and plant height (cm) (bottom row) in the 2012 dry season, using 10 selections of SNP subsets either distributed evenly throughout the genome (left column) or chosen at random (right column) and five different statistical methods, error bars constructed using 1 standard error from the mean. The training population consisted of data from years 2009–2011, both seasons per year.
