# Supplementary material for: Genomic Selection and Association Mapping in Rice (Oryza sativa): Effect of Trait Genetic Architecture, Training Population Composition, Marker Number and Statistical Model on Accuracy of Rice Genomic Selection in Elite, Tropical Rice Breeding Lines
Source: PLoS Genet. 2015 Feb 17;11(2):e1004982. doi: 10.1371/journal.pgen.1004982 (PMC4334555; doi:10.1371/journal.pgen.1004982)
Supplement: S1 Table — (DOCX) [file pgen.1004982.s006.docx]

Table S1. Summary of missing data for the remaining 332 genotyped lines (after outlier removal) by year and season.

| Year | Season | lines with data | lines missing |
| --- | --- | --- | --- |
| 2009 | dry | 114 | 218 |
| 2009 | wet | 163 | 169 |
| 2010 | dry | 166 | 166 |
| 2010 | wet | 209 | 123 |
| 2011 | dry | 327 | 5 |
| 2011 | wet | 328 | 4 |
| 2012 | dry | 325 | 7 |
| 2012 | wet | 324 | 8 |
